# Longitudinal Health Risk Assessment of Neonicotinoid Exposure and Its Association with Dietary Sources in School-Aged Children: A Prospective Cohort Study

**DOI:** 10.3390/toxics13121058

**Published:** 2025-12-05

**Authors:** Boya Zhang, Yiming Dai, Jiming Zhang, Zheng Wang, Jiayun Ding, Xingzu Zhou, Xiaojuan Qi, Zhijun Zhou

**Affiliations:** 1 MOE Key Laboratory of Public Health Safety, NHC Key Laboratory of Health Technology Assessment, School of Public Health, Fudan University, No. 130 Dong’an Road, Shanghai 200032, China; 22111020059@m.fudan.edu.cn (B.Z.); 20111020031@fudan.edu.cn (Y.D.); 23111020063@m.fudan.edu.cn (Z.W.); 22211020077@m.fudan.edu.cn (J.D.); 24211020091@m.fudan.edu.cn (X.Z.); xjqi@cdc.zj.cn (X.Q.); 2 Zhejiang Provincial Center for Disease Control and Prevention, No. 3399 Binsheng Road, Hangzhou 310051, China

**Keywords:** neonicotinoid insecticides, longitudinal study, school-aged children, health risk assessment, dietary sources

## Abstract

Neonicotinoid insecticides (NNIs) are globally pervasive, and toxicological evidence indicates potential adverse effects from low-dose exposure in non-targeted organisms. Humans may be exposed to NNIs through multiple pathways, such as ingestion and inhalation, with dietary intake recognized as the dominant exposure route. However, longitudinal evidence characterizing evolving exposure patterns in rural children remains scarce. We evaluated temporal trends and dietary determinants of NNI exposure among 643 children at ages 7, 10, and 14 years in the Sheyang Mini Birth Cohort Study. Twelve NNIs and six metabolites in urine samples were measured using UPLC-HRMS; estimated daily exposure doses and hazard index (HI) were calculated, and linear mixed models were used to evaluate dietary determinants of NNI exposure. Widespread exposure was observed (ΣNNIs detection: 98.8–100%), and although cumulative risks remained below safety thresholds, both medians and upper bounds of HI increased with age (0.0007 to 0.0074; 0.2045 to 0.4054). Notably, exposure composition shifted, with declining imidacloprid and emerging dominance of clothianidin (CLO) and thiamethoxam (THM). Fruit and vegetable intakes were positively associated with ΣNNIs, whereas cereals, poultry, and eggs showed inverse associations, with more pronounced effects observed in boys. These findings indicated persistent yet evolving exposure risks in school-aged children, highlighting fruits and vegetables as major contributors. Although current toxicological risk appears low, the transition toward CLO and THM—compounds with limited chronic toxicity data—underscores the need for continued biomonitoring and targeted exposure mitigation.

## 1. Introduction

Neonicotinoid insecticides (NNIs), an emerging class of synthetic pesticides, have been widely used to protect crops, livestock, and pets from pests [1], since NNIs could act selectively on insect nicotinic acetylcholine receptors (nAChRs) and induce overstimulation of cholinergic synapses, resulting in paralysis, convulsions, and ultimately death [2]. Owing to their high selectivity and broad-spectrum insecticidal efficacy, NNIs were initially hailed as a milestone in insecticide development [3]. Since the commercial introduction of imidacloprid (IMI) in 1991, NNIs have rapidly captured over a quarter of the global insecticide market [4], yet their widespread application has raised concerns about environmental pollution and potential human health risks [5]. Humans can be exposed to NNIs through ingestion, inhalation, or even dermal contact, and dietary intake has been recognized as the primary source [5,6]. The systemic nature of NNIs enables translocation within plant tissues, leading to persistent residues in edible portions [7,8]. In addition, Chinese total diet studies (TDSs) revealed a substantial increase in NNI contamination, with detection frequencies rising from 53.3% in the 5th TDS (2009–2012) to 70.5% in the 6th TDS (2015–2018) [9]. These findings underscore the critical need to evaluate dietary exposure risks associated with NNIs.

Children are known as particularly vulnerable to environmental chemicals like NNIs due to their physiological susceptibility and exposure-prone behaviors [10,11]. During the critical developmental period, children have higher dietary intake per kg of body weight than adults, and biological barriers and detoxification systems are not yet well developed to eliminate xenobiotics. In addition, child-specific behaviors like hand-to-mouth contact increase dermal and oral exposure [12]. Several epidemiological studies linked NNI exposure to multiple pediatric health outcomes, including neurodevelopmental defects in toddlers [13], overweight and obesity in schoolchildren [14], and sex and adrenal hormone disruption in adolescents [15]. Toxicological evidence also supported potential adverse effects from non-acute NNI exposure. As summarized in a recent article, repeated low-dose exposure to commonly used NNIs induced hepatotoxicity, neurobehavioral alterations, oxidative stress, and reproductive effects in rodents [16]. Another recent systematic review also emphasized that chronic, time-cumulative toxicity may arise even at doses far below acute LD_50_ values, highlighting the risk of long-term and low-dose NNI exposure in mammals [17]. Nevertheless, current risk assessments remain inadequate to safeguard children.

Despite accumulating research on NNI exposure risks across populations, critical knowledge gaps persist in characterizing long-term exposure patterns. First, the rapid evolution of NNI compounds poses challenges. As evidenced by the phasing out of IMI due to its demonstrated adverse effects on non-target organisms and subsequent restrictions in several countries [18], newer alternatives like sulfoxaflor (SUL) and cycloxaprid (CYC) have been increasingly adopted [8], whose toxicity remains poorly characterized. Second, significant regional variations exist in exposure profiles, driven by differing crops and NNI usage [19,20]. For instance, a Chinese biomonitoring study revealed distinct urinary metabolite profiles, in which dinotefuran (DIN) metabolites were the dominant compounds in samples from Hebei and Shandong provinces, and 5-hydroxy-imidacloprid (5-OH-IMI) and N-desmethyl-acetamiprid (N-dm-ACE) metabolites were the most prevalent metabolites in Jiangsu and Guangdong provinces, respectively. Furthermore, dietary exposure to NNIs is a chronic, long-term, and cumulative process; risk assessments at a single point are insufficient to capture comprehensive health risks [9].

In this study, all participants lived in Sheyang County, Jiangsu Province, situated along the eastern coast of China. The region has a subtropical monsoon climate and primarily cultivates rice, wheat, rapeseed, and cotton. The objective of this study is to assess the long-term health risk and patterns of NNI exposure among local school-aged children and to identify the major dietary sources contributing to NNI exposure.

## 2. Method and Materials

### 2.1. Study Design and Study Population

The study population came from the ongoing Sheyang Mini Birth Cohort Study (SMBCS), a prospective cohort study focusing on the environmental chemical exposure and child health. The detailed information of SMBCS was first described in our previous publication [21]. In brief, 1303 mother-child pairs were recruited between June 2009 and January 2010. Subsequently, as children reached ages 7, 10, and 14 years old, 454, 499, and 360 mother-child pairs were repeatedly followed up in 2016, 2019, and 2023, respectively. In each follow-up, caregivers reported on demographic and socioeconomic information using standardized questionnaires. The 24 h dietary recall was provided by children with assistance from their caregivers and researchers. Single-spot urine samples of children were collected by trained researchers. The height and weight of children were measured by local pediatricians with an accuracy of 0.1 cm and 0.01 kg, respectively. Body mass index (BMI, kg/m^2^) was calculated as weight/(height^2^). BMI z-scores were derived based on the WHO Growth Reference for Children and Adolescents Aged 5–19 years (2007). According to this reference, overweight and obesity were defined as BMI z-scores ≥ +1 standard deviation (SD) and ≥+2 SD, respectively [22]. Finally, we restricted the study population to children who participated in at least one follow-up at their 7, 10, and 14 years. A total of 643 children were included in this study, in which 411, 485, and 356 children had completed data on urinary NNIs and creatinine and 24 h dietary survey at 7, 10, and 14 years of age, respectively. The research protocol was approved by the Ethics Committee of the School of Public Health, Fudan University (IRB#2021-02-0875-S). All participants (both children and their caregivers) were informed and signed the informed consent.

### 2.2. Measurement of NNIs in Urine

Urine samples were collected in high-density polypropylene centrifuge tubes (Corning Incorporated, Corning, NY, USA) and stored at −80 °C until analysis. Based on the method we developed previously [23], 12 NNIs and 6 metabolites were simultaneously measured, including acetamiprid (ACE), clothianidin (CLO), CYC, DIN, flonicamid (FLO), flupyradifurone (FLU), IMI, imidaclothiz (IMID), nitenpyram (NIT), thiacloprid (THIA), thiamethoxam (THM), SUL, N-dm-ACE, 1-methyl-3-(tetrahydro-3-furylmethyl) guanidine (DIN-G), 1-methyl-3-(tetrahydro-3-furylmethyl) urea (DIN-U), 5-OH-IMI, 6-chloronicotinic acid (6-CNA), and olefin-imidacloprid (Of-IMI) (Table S1). Isotope-labeled internal standards were used for quantification, including acetamiprid-d_3_, dinotefuran-d_3_, imidacloprid-d_4_, thiamethoxam-d_3_, and clothianidin-d_3_. Briefly, 200 μL of urine was acidified and enzymatically incubated overnight, then the supernatant was taken for solid-phase extraction without washing. The elution solvent was evaporated by nitrogen blowing, then re-dissolved in 50 μL of 5% methanol for instrument analysis. The ultra-high-performance liquid chromatography-quadrupole/orbitrap high-resolution mass spectrometry (UPLC-Q/Orbitrap HRMS, ThermoFisher, Waltham, MA, USA) was used for compound separation and analysis. Three levels of quality control (QC) samples (0.4, 1.6, and 4.0 ng/mL) were prepared by 10-fold water-diluted mixed urine. Solvent blanks, process blanks, QC samples, and 10% duplicate samples were added in each batch to evaluate the accuracy and precision. For most analytes, the calibration range was 0.1–10 ng/mL with seven calibration points; for DIN and Of-IMI, the range was 0.5–10 ng/mL; and for SUL-α and SUL-β, the lowest point was 1.0 ng/mL. The limit of detection (LOD) for NNIs ranged from 0.01 μg/L to 0.65 μg/L, with the relative recoveries of the methods ranging from 81.6% to 122.4%. Urinary creatinine concentration was measured by the Elx800 Universal Microplate Reader (Biotech Instruments Inc., Winooski, VT, USA) and the sarcosine oxidase assay kit (Jiancheng, Nanjing, China).

### 2.3. Estimation of Daily Exposure Dose (EDED)

As shown in Table S2, we estimated the daily exposure dose of NNIs for each child by Equation (1), which was commonly used to estimate the daily exposure dose of non-persistent environmental pollutants [24,25]:

(1) $$EDED=\left(Cn\times Mc\right)/\left(Cc\times Mb\times P\times 1000\right)$$

Cn and Cc were the neonicotinoid concentration (ng/mL) and creatinine concentration (mg/mL) in urine, respectively. Mb was the body weight (kg) of the child. Mc means daily output of creatinine in urine (mg/day), which was predicted by Equation (2) [26], and H was body height (cm):

(2) $$Mc=113.1\times 10^{\left(0.0102\times H-0.6854\right)}$$

P means the NNI excretion proportion in urine as unchanged or metabolite forms [25]. Based on the human pharmacokinetic data, the excretion proportion of CLO, IMI, DIN, and N-dm-ACE in urine was 0.596, 0.133, 0.899, and 0.586 [27], and ACE, FLU, SUL, 5-OH-IMI, 6-CNA, and Of-IMI in urine were 0.48, 0.33, 0.51, 0.31, 0.072, and 0.27 [28]. Due to a lack of human pharmacokinetic data, the urine excretion proportions of CYC (0.745) [29] and THIA (0.63) in rats [30], and NIT (0.46) and THM (0.75) in mice [31], were used instead. For other NNIs/metabolites, since no human or animal pharmacokinetic data were available, their urine excretion proportions were set to those of compounds with a similar chemical structure or parent compound [31]. Specifically, the excretion proportion of FLO was 0.51, which was the same as SUL; that of IMID was 0.133, the same as IMI; and DIN-G and DIN-U were both 0.899, the same as their parent compounds—DIN.

### 2.4. Health Risk Assessment

The health risk of individual NNI exposure was assessed as the hazard quotient (HQ), which was calculated as the ratio of EDED to the health safety threshold [31]. Considering that NNIs adversely affect humans in the same mode of action, we used the hazard index (HI), the arithmetic sum of HQ, to assess the cumulative health risk of combined exposure to NNIs [32]. The health safety threshold in this study referred to the chronic reference dose (cRfD) proposed by the Environmental Protection Agency (EPA) of the United States or the acceptable daily dose (ADI) proposed by the Ministry of Agriculture (MOA) of China (Pesticide acceptable daily intake, NY/T 2874–2015) [33]. If both cRfD and ADI are available, the lower one is used. If cRfDs were lacking, ADIs (mg/kg·d) were used as the safety thresholds for FLO (0.07), FLU (0.08), IMID (0.025), NIT (0.53), and SUL (0.05). HQ > 1 or HI > 1 indicates a potential health risk. CYC and NNI metabolites were not included in the calculation of HQ and HI due to the lack of health safety thresholds for them (Table S2).

### 2.5. Dietary Assessment

Twenty-four-hour dietary recall was used to collect the dietary intake and detailed information on the food consumption of children. With the help of the caregivers, trained investigators interviewed the child face-to-face about what they had eaten in the past 24 h. According to the Chinese Food Composition Tables (CFCT) 2018 (National Institute of Nutrition and Food Safety, China CDC), we divided foods into 20 groups and calculated the intake weight (g) for each food group. Since some food groups were under-consumed by the children in this study, only eight food groups, including cereals, vegetables, fruits, meat and meat products, poultry and poultry products, dairy products, eggs and egg products, and aquatic products, were retained for subsequent analysis (Table S3).

### 2.6. Statistical Analysis

The sociodemographic characteristics of children at 7, 10, and 14 years were summarized using mean (±standard deviation) or number (percentile, %). To better represent NNI exposure, the creatinine-adjusted concentration [μg/g Cr (μg/g creatinine)] of the p-NNIs and their specific metabolite(s), in units of nmol/g creatinine (nmol/g Cr), were summed, generating ΣACE (ACE + N-dm-ACE), ΣDIN (DIN + DIN-G + DIN-U), ΣIMI (IMI + 5-OH-OMI + Of-IMI), and ΣNNIs (total sum of p-NNIs and m-NNIs). A positive detection of the sum of NNIs in one urine sample was defined as a positive detection of at least one compound in this group. The detection frequency, percentile, and maximum were used to describe the distribution of creatinine-adjusted urinary NNI concentrations [μg/g Cr (μg/g creatinine)]. Box plots with scatters were used to present the distribution of EDED, HQ, and HI of detected urinary NNIs in children at 7, 10, and 14 years. Urinary NNIs were log_2_-transformed to approximate a normal distribution, and concentrations below LOD were replaced by half of the minimum.

Further analyses were restricted to the sum of NNIs with detection frequencies higher than 60%. Pearson correlation coefficients were calculated to reflect the correlations between log_2_-transformed NNI concentrations in each year. A linear mixed model (LMM) was employed to evaluate the longitudinal association between NNI exposure and dietary intake in school-aged children. Potential covariates were selected based on prior literature and statistical considerations. If the covariate was related to urinary NNIs concentrations or changed the coefficients of NNIs concentrations by more than 10%, we included it in the model. Finally, LMM were adjusted for sex (boys or girls), maternal education (under high school or above or equivalent to high school), maternal occupational type (mental work, manual work, or other), residence (suburb, town, or countryside), annual household income (≤30,000 CNY or >30,000 CNY), passive smoking (yes or no), and childhood BMI z-score (continuous variable). In consideration of sex-specific effects, we included an interaction term (sex × dietary) in LMM, and sex-stratified analyses were employed when *p_sex-int_* < 0.2. To address potential follow-up bias and assess the robustness of models, we conducted a sensitivity analysis by re-estimating the LMM using only participants with complete longitudinal data at ages 7, 10, and 14 years. Considering the varied detection frequencies of some NNIs across age groups, multivariable linear models were used to evaluate the associations between dietary food groups and NNIs with high detection frequencies (>60%) at each single time point.

## 3. Results

### 3.1. Characteristics of the Study Population

Table 1 demonstrates the sociodemographic characteristics of the study population across follow-up periods. The sex distribution remained balanced among all age groups (boys: 51.7–56.0%; girls: 44.0–48.3%, *p* = 0.472). Among the three follow-ups, notable socioeconomic transitions were observed. Specifically, the proportion of mothers with higher education attainment increased significantly from 29.4% at age 7 to 41.6% at age 14 (*p* < 0.001), while mothers with manual work predominated at ages 10 and 14 years (62.5% and 63.5%). Household income exhibited marked improvement, with over 86% reporting annual incomes exceeding 30,000 CNY at ages 10 and 14 years compared to 55.7% at age 7 (*p* < 0.001). The passive smoking status in children decreased significantly at 14 years, with only 17.4% (*p* < 0.001). Notably, mean BMI z-scores exhibited a significant upward trend across age groups, increasing from 0.3 at 7 years to 0.6 at 14 years (*p* = 0.002), and the prevalence of obesity more than doubled from 10.0% to 20.5% over the same period (*p* < 0.001).

### 3.2. Urinary NNI Concentrations

Table 2 presents creatinine-adjusted urinary NNIs concentrations in children at ages 7, 10, and 14 years. ΣNNI exposure was nearly ubiquitous, with detection frequencies of 98.76–100% across all time points. Among p-NNIs, ACE, DIN, FLU, IMID, THIA, and ∑SUL remained low detection frequencies (<20%), while CLO and THM demonstrated age-dependent increases in detection frequencies (CLO: 30.66% at 7 years to 77.81% at 14 years; THM: 24.09% to 52.53%). For m-NNIs, N-dm-ACE was the predominant metabolite, detected in above 95% of samples with median concentrations of 0.82 to 0.91 μg/g Cr. ΣACE exhibited consistently high detection (>96%) and stable median concentrations (3.93–4.47 nmol/g). Conversely, ΣIMI showed a declining trend, with detection frequencies decreasing from 48.42% to 17.70%, and 75th percentile concentrations dropping from 9.57 nmol/g at 7 years to below the LOD by adolescence. ΣDIN showed a fluctuating trend, reaching the lowest median concentrations at 10 years (1.28 nmol/g, detection frequency: 61.86%), which was approximately half the values observed at 7 years (2.33 nmol/g) and 14 years (2.52 nmol/g). There was no significant correlation between ΣACE and ΣDIN within the same time and across times, and only a weak correlation was observed between ΣNNIs at 10 years and 14 years (r = 0.13) (Figure S1).

### 3.3. Estimation of Daily Exposure Dose and Health Risk of NNIs

**Figure 1:**
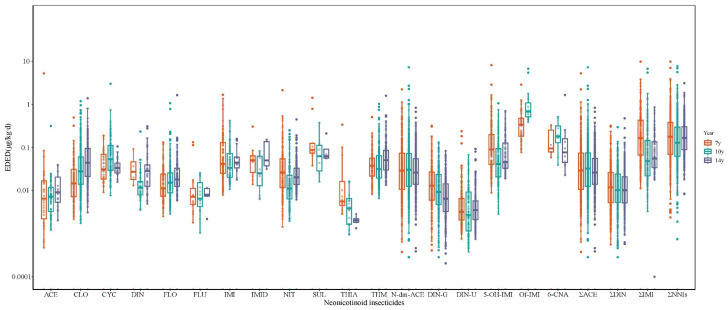
Estimation of daily exposure dose (EDED; μg/kg·d) of neonicotinoids in children. ACE, acetamiprid; CLO, clothianidin; CYC, cycloxaprid; DIN, dinotefuran; FLO, flonicamid; FLU, flupyradifurone; IMI, imidacloprid; IMID, Imidaclothiz; NIT, nitenpyram; SUL, sulfoxaflor; THIA, thiacloprid; THM, thiamethoxam; N-dm-ACE, N-desmethyl-acetamiprid; DIN-G, 1-methyl-3-(tetrahydro-3-furylmethyl) guanidine; DIN-U, 1-methyl-3-(tetrahydro-3-furylmethyl) urea; 5-OH-IMI, 5-hydroxy-imidacloprid; 6-CNA, 6-chloronicotinic acid; Of-IMI, olefin-imidacloprid; ∑ACE, the molar sum of ACE and N-dm-ACE; ∑DIN, the molar sum of DIN, DIN-G, and DIN-U; ∑IMI, the molar sum of IMI, 5-OH-IMI, and Of-IMI; ∑NNIs, the molar sum of all neonicotinoids.

The maximum of EDED of ΣNNIs declined from 9.803 μg/kg/day at 7 years to 3.096 μg/kg/day at 14 years (Figure 1, details shown in Table S4). Median EDEDs for ΣACE and ΣDIN remained stable (0.026–0.030 and 0.004–0.008 μg/kg/day, respectively), whereas ΣIMI exposure demonstrated a pronounced decline: its 75th percentile and maximum EDED decreased from 0.156 μg/kg/day and 9.770 μg/kg/day at 7 years of age to below LOD and 0.866 μg/kg/day at 14 years of age. Conversely, the EDED maximum for CLO, FLO, and THM increased with age, reaching 1.381, 1.637, and 1.586 μg/kg/day, respectively.

**Figure 2:**
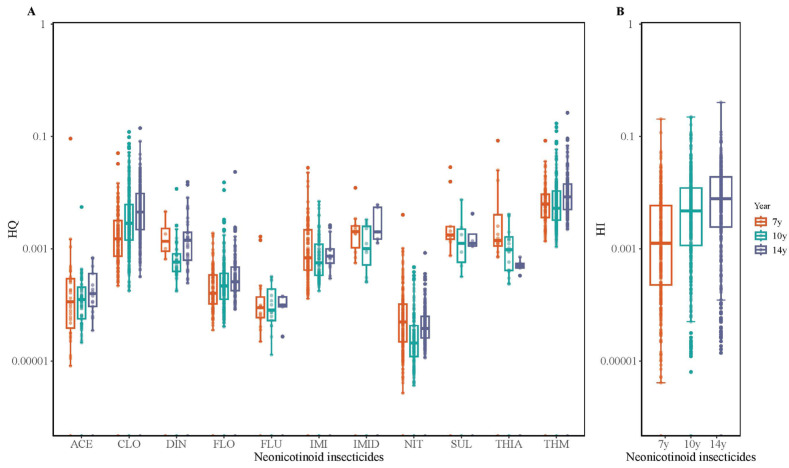
Health risk assessment of neonicotinoids in children by hazard quotient (HQ; (**A**)) and hazard index (HI; (**B**)). ACE, acetamiprid; CLO, clothianidin; DIN, dinotefuran; FLO, flonicamid; FLU, flupyradifurone; IMI, imidacloprid; IMID, Imidaclothiz; NIT, nitenpyram; THIA, thiacloprid; THM, thiamethoxam.

Both HQ and HI remained below the safety threshold of 1 across all ages (Figure 2, details shown in Table S5). The ranges of maximum HQs at ages of 7, 10, and 14 years were 0.0017–0.0916, 0.0003–0.1710, and 0.0007–0.2643. THM showed the highest HQ at the ages of 10 and 14 years (0.1710 and 0.2643), followed by CLO (0.1210 and 0.1410). Median HI increased from 0.0007 at age 7 to 0.0074 at age 14, while maximum HI rose from 0.2045 to 0.4054.

### 3.4. Association of Dietary Food Groups and Childhood NNI Exposure

**Figure 3:**
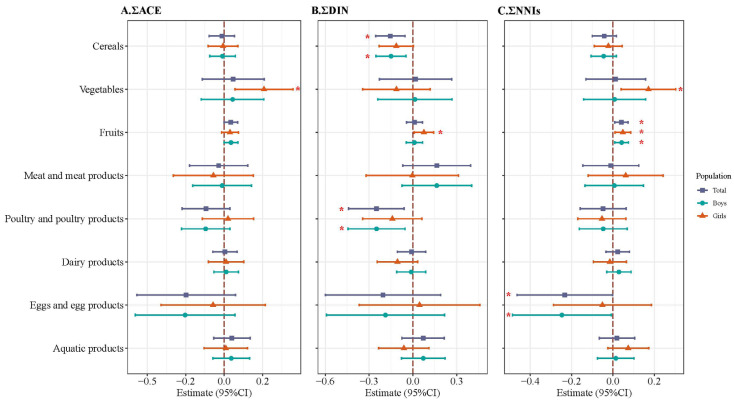
Associations between dietary sources and urinary neonicotinoid insecticides among school-aged children using linear mixed models. Models were adjusted by sex, maternal education, maternal occupational type, residence, annual household income, passive smoking, and childhood BMI z-score. (**A**) ∑ACE, the molar sum of ACE and N-dm-ACE; (**B**) ∑DIN, the molar sum of DIN, DIN-G, and DIN-U; (**C**) ∑NNIs, the molar sum of all neonicotinoids. * means *p*-value < 0.05.

Figure 3 (details shown in Table S6) presents the associations between dietary intake (per 100 g increment) and log_2_-transformed urinary NNI concentrations via LMM. Fruit intake was positively associated with ΣNNIs, corresponding to a 0.041 unit increase per 100 g increment (β = 0.041, 95%CI: 0.009, 0.073). Conversely, eggs and egg products were negatively associated with ΣNNI exposure (β = −0.233, 95%CI: −0.464, −0.002). Cereal and poultry products were linked to reduced ΣDIN level (β = −0.155, 95%CI: −0.255, −0.054; β = −0.250, 95%CI: −0.441, −0.060). Significant sex-interaction terms were observed for vegetable intake with ΣACE and ΣNNIs (*p_sex_*_-int_ = 0.107; *p_sex_*_-int_ = 0.089) and for fruit intake with ΣDIN (*p_sex_*_-int_ = 0.123). After sex stratification, the positive associations between vegetable intake and both ΣACE and ΣNNIs were only significant in girls (β = 0.259, 95%CI: 0.070, 0.448; β = 0.172, 95%CI: 0.040, 0.304). Similarly, fruit intake was associated with increased ΣDIN exposure exclusively in girls (β = 0.075, 95%CI: 0.009, 0.142). In addition, the negative associations of cereal and poultry products with ΣDIN (β = −0.150, 95%CI: −0.253, −0.047; β = −0.249, 95%CI: −0.444, −0.054) and of egg products with ΣNNIs (β = −0.247, 95%CI: −0.486, −0.008) were only observed in boys. The results of LMM in children who participated in all three follow-ups (N = 199) were consistent with results mentioned above (Table S7). No significant associations were observed between dietary food groups and CLO and NIT in GLM (Table S8).

## 4. Discussion

In the current study, urinary NNIs were detected in school-aged children from SMBSC. The EDED and HI values at ages 7, 10, and 14 years suggested that children living in Sheyang County may not have any potential risk associated with NNI exposure. The intakes of vegetables and fruits were associated with higher NNI exposure, while the intakes of cereals, poultry and eggs, and their products were negatively associated with NNI exposure. Continuous monitoring of NNI exposure is warranted owing to the increasing trend of HI, changes in exposure patterns, as well as dietary intake risks.

The urinary profiles and concentrations of NNIs exhibited substantial regional and temporal variations among children (Table S9). Compared to previous studies, our SMBCS generally demonstrated lower NNI concentrations and detection frequencies. Although China accounts for approximately 14,000 tons of annual IMI production [34]—ranking as the global leader in NNI usage—our findings (∑IMI: 48.42% in 2016; 40.21% in 2019) contrast with three contemporaneous Chinese studies (2017–2020) reporting significantly higher detection frequencies of IMI in Shenzhen (95.7%) [35], Shandong (99.7%) [36], and Chongqing (99.8%) [37]. The detection frequency of N-dm-ACE in our 10-year follow-up (96.08%) aligned with that in Shandong (100.0%) [36] and Chongqing (98.0%) [37], but Chongqing’s creatinine-adjusted median concentration (34.27 μg/g Cr) exceeded ours (0.91 μg/g Cr) by over 30-fold [37]. Similarly, Shanghai children exhibited markedly lower detection frequencies (<10% for most NNIs), except for clothianidin (CLO, 53.6%) and N-dm-ACE (57.0%) [14]. Cross-national comparisons further revealed distinct exposure patterns. During overlapping sampling periods (2015–2016), N-dm-ACE dominated in our 7-year-old children (96.35%), whereas Japanese toddlers showed high DIN exposure (45.8%) [38], and U.S. adolescents exhibited high 5-OH-IMI detection (20.1%) [39]. Importantly, our longitudinal data revealed increasing trends of CLO and THM exposure across three follow-ups, consistent with the findings of two consecutive TDS in China. Specifically, the detection frequencies of CLO rose from 9.6% (5th TDS, 2009–2012) to 52.8% (6th TDS, 2015–2018), paralleled by THM’s increase from 22.9% to 54.9% [9]. These regional disparities may be attributable to differences in local agricultural practices, climatic and geographical environment, and dietary preferences. More longitudinal biomonitoring studies are warranted to assess the exposure patterns and potential risk of NNIs in specific regions. In addition, differences in analytical techniques and laboratory detection limits across studies may also contribute to the observed disparities, although our use of high-resolution UPLC-Q/Orbitrap HRMS with low LODs minimizes the likelihood of under-detection within our cohort.

The NNI exposure in our study showed relatively lower levels and dynamic temporal shifts. The median EDED of ∑NNIs (0.127–0.177 μg/kg/d) was similar to that in Shanghai children (0.13 μg/kg/d) [32], but was much lower than values in Japanese children living in pine wilt disease control areas (before, during, and after insecticide spraying exercise: 1.02, 1.26, and 1.13 μg/kg/d) [40]. In addition, the maximum EDED of ∑NNIs declined from 9.803 μg/kg/d to 3.096 μg/kg/d, suggesting effective mitigation of extreme exposures, potentially due to improved protective measures in farming households or heightened public awareness of pesticide residues. Notably, exposure patterns further revealed a significant decline in ∑IMI (75th percentile at 7, 10, and 14 years: 0.156 μg/kg/d, 0.033 μg/kg/d, and <LOD), followed by rising exposures to CLO (0.006, 0.039, and 0.078 μg/kg/d) and THM (<LOD, 0.031, and 0.052 μg/kg/d). These shifts mirror residue trends in China’s TDS, where IMI detection in cereals decreased (85% to 62.5%), while CLO (30% to 70.8%) and THM (30% to 62.5%) increased between the 5th and 6th surveys [9]. Such transitions may be associated with agricultural practices in Sheyang County, a major producer of rice and wheat crops increasingly treated with CLO and THM as alternatives to phased-out IMI [41]. Health risk assessment indicated low but rising cumulative risks in Chinese school-aged children, primarily driven by CLO and THM. Despite their moderate exposure levels, the cRfD of CLO (0.0098 mg/kg/day) and THM (0.006 mg/kg/day), much lower than those of other NNIs (e.g., ADI of IMI: 0.057 mg/kg/day), lead to a higher health risk. CLO was demonstrated to have comparable chronic toxicity to IMI in *Chironomus dilutus*, and THM can readily degrade to CLO under field conditions to enhance toxicity [42]. These findings highlight the necessity of prioritizing CLO and THM in further biomonitoring programs.

LMM revealed significant associations of dietary groups with NNI exposure in childhood. Consistent with findings in pregnant women [31], elevated fruit and vegetable consumption was linked to higher urinary NNI levels, likely due to prolonged cultivation cycles, intensive insecticide use, and the systemic absorption properties of NNIs in edible tissues. In the Yangtze River Delta, China, NNIs were widely used, and residues varied among different croplands, with the highest level detected in fruit fields, followed by vegetables [43]. Vegetable samples collected in the 5th and 6th TDS showed the highest frequencies of detection for the multi-residue of NNIs [9], and cleaning and peeling have a limited effect on NNI residue removal due to their systemic penetration into the plant tissue [44]. Of note, the positive association between vegetables and NNI exposure was only observed in girls, which may be due to their greater adherence to the dietary pattern characterized by the higher consumption of vegetables than boys [45]. Negative associations between cereals, poultry products, and eggs were more pronounced in boys, which seemed paradoxical considering that cereals, including wheat, rice, and corn, as components of poultry’s fodder, are the primary potential source of neonicotinoid contamination [46]. One possible explanation is the degradation or evaporation of NNIs during high-temperature processing like steaming, boiling, and frying [47], and boys prefer those processed foods. Sex-specific toxicokinetic differences, including hormonal regulation of nuclear receptors to hormones and expression patterns of cytochrome P450 enzymes [48], may further modulate these exposure disparities.

This longitudinal study leveraged repeated biomonitoring at ages 7, 10, and 14 years to evaluate long-term health risks and dynamic shifts in NNI exposure patterns among school-aged children while exploring associations between dietary intake and NNI levels. However, some limitations warrant acknowledgment. First, the relatively small sample size and the single-site design in Sheyang County may limit generalizability to urban or non-agricultural populations. Second, the 24 h dietary recall did not include information on food preparation methods such as washing, peeling, or frying, which may substantially alter pesticide residue levels and thus introduce exposure misclassification. Moreover, a single 24 h recall may not accurately represent children’s usual dietary patterns, potentially attenuating true associations. Third, reliance on animal-derived pharmacokinetic data for certain NNIs (e.g., CYC) introduces uncertainty in exposure estimates. Fourth, although the 24 h dietary recall allowed us to examine associations between dietary patterns and internal NNI levels, the study did not include direct measurements of NNI residues in foods. The EDED derived from Equation (1) should be interpreted as an approximate internal-dose indicator rather than a precise estimate of absolute intake. Fifth, urinary NNIs were creatinine-adjusted to correct for dilution, but creatinine levels varied by age, gender, muscle mass, and so on [49], which may result in exposure misclassification. Finally, non-dietary exposures (e.g., environmental contamination) were not assessed. Future multi-regional cohorts with larger sample sizes and comprehensive exposure source evaluations are needed to validate and extend these findings.

## 5. Conclusions

In conclusion, school-aged children in rural China were ubiquitously exposed to NNIs. Although cumulative health risks remained below established safety thresholds, both median and upper-bound hazard indices increased steadily with age, indicating a gradual rise in NNI exposure across childhood. Temporal shifts in exposure patterns were evident, characterized by declining IMI levels and rising CLO and THM levels. Longitudinal analyses further indicated fruit and vegetable consumption was the primary dietary source of NNIs in school-aged children, whereas cereals, poultry, and eggs showed inverse associations, with notable sex-specific differences. These findings highlighted the evolving nature of NNI exposure during childhood and underscore the importance of continued biomonitoring and targeted mitigation strategies focusing on dietary pathways.

## Figures and Tables

**Table 1:** Sociodemographic characteristics of study population [mean ± standard deviation or number (percentile, %)].

Characteristics	7 Years (N = 411)	10 Years (N = 485)	14 Years (N = 356)	*p*-Value
Sex				0.472
Boys	230 (56.0)	256 (52.8)	184 (51.7)	
Girls	181 (44.0)	229 (47.2)	172 (48.3)	
Maternal education				**<0.001**
Under high school	290 (70.6)	349 (72.0)	208 (58.4)	
Above or equivalent high school	121 (29.4)	136 (28.0)	148 (41.6)	
Maternal occupation type				**<0.001**
Mental work	107 (26.1)	127 (26.2)	81 (22.8)	
Manual work	195 (47.4)	303 (62.5)	226 (63.5)	
Other	109 (26.5)	55 (11.3)	49 (13.8)	
Annual household income				**<0.001**
≤30,000 CNY	182 (44.3)	67 (13.8)	48 (13.5)	
>30,000 CNY	229 (55.7)	418 (86.2)	308 (86.5)	
Residence				0.745
Suburb	130 (31.6)	154 (31.8)	118 (33.1)	
Town	92 (22.4)	97 (20.0)	73 (20.5)	
Countryside	189 (46)	234 (48.2)	165 (46.3)	
Passive smoking				**<0.001**
Yes	137 (33.3)	234 (48.2)	62 (17.4)	
No	274 (66.7)	251 (51.8)	294 (82.6)	
Body Mass Index z-score (BMI z-score)	0.3 ± 1.3	0.6 ± 1.4	0.6 ± 1.3	**0.002**
Non-overweight or obese	310 (75.4)	303 (62.5)	215 (60.4)	**<0.001**
Overweight	60 (14.6)	98 (20.2)	68 (19.1)	
Obese	41 (10.0)	84 (17.3)	73 (20.5)	

Note: Differences in categorical variables were assessed using Pearson’s chi-square test, and continuous variables were assessed using one-way ANOVA with Levene’s test for homogeneity of variance. Bold consent means *p*-value < 0.05.

**Table 2:** The distribution of creatinine-adjusted urinary neonicotinoid concentrations among children at 7, 10, and 14 years.

Analytes	7 Years (n = 411)			10 Years (n = 485)			14 Years (n = 356)		
	≥LOD (%)	Median (25th, 75th, 95th)	Max	≥LOD (%)	Median (25th, 75th, 95th)	Max	≥LOD (%)	Median (25th, 75th, 95th)	Max
p-NNIs (μg/g)									
ACE	7.54	<LOD (<LOD, <LOD, 0.09)	134.6	14.02	<LOD (<LOD, <LOD, 0.22)	5.11	4.78	<LOD	0.82
CLO	30.66	<LOD (<LOD, 0.2, 1.46)	18.32	65.15	0.43 (<LOD, 1.33, 7.83)	32.19	77.81	0.98 (0.21, 2.37, 6.51)	34.58
CYC	8.76	<LOD (<LOD, <LOD, 1.2)	7.44	30.93	<LOD (<LOD, 0.97, 7.07)	109.01	6.74	<LOD (<LOD, <LOD, 0.98)	3.98
DIN	0.97	<LOD	5.29	8.66	<LOD (<LOD, <LOD, 0.44)	10.06	10.39	<LOD (<LOD, <LOD, 1.12)	12.89
FLO	29.44	<LOD (<LOD, 0.16, 0.79)	3.77	44.33	<LOD (<LOD, 0.38, 1.14)	26.48	33.43	<LOD (<LOD, 0.35, 1.16)	38.77
FLU	3.65	<LOD	2.28	3.51	<LOD	0.55	1.40	<LOD	0.23
IMI	28.95	<LOD (<LOD, 0.15, 1.18)	11.38	16.7	<LOD (<LOD, <LOD, 0.48)	2.74	4.78	<LOD	1.57
IMID	2.43	<LOD	2.12	2.27	<LOD	0.76	1.40	<LOD	0.99
NIT	45.26	<LOD (<LOD, 0.58, 2.48)	61.67	31.96	<LOD (<LOD, 0.14, 0.84)	5.81	73.88	0.39 (<LOD, 0.5, 1.40)	8.18
THIA	2.92	<LOD	11.43	4.12	<LOD	0.76	1.40	<LOD	0.12
∑SUL	2.19	<LOD	38.87	1.24	<LOD	11.31	1.12	<LOD	5.09
THM	24.09	<LOD (<LOD, <LOD, 2.47)	20.66	48.66	<LOD (<LOD, 1.30, 4.55)	42.57	52.53	0.79 (<LOD, 1.96, 5.79)	49.96
m-NNIs (μg/g)									
N-dm-ACE	96.35	0.88 (0.31, 2.24, 9.99)	61.45	96.08	0.91 (0.36, 2.22, 7.42)	273.86	99.16	0.82 (0.43, 1.76, 5.46)	22.3
DIN-G	54.99	0.1 (<LOD, 0.73, 2.83)	17.65	49.28	<LOD (<LOD, 0.46, 2.09)	7.16	71.63	0.19 (<LOD, 0.49, 1.42)	3.48
DIN-U	46.47	<LOD (<LOD, 0.15, 0.62)	12.99	20.41	<LOD (<LOD, <LOD, 0.42)	4.58	70.51	0.12 (<LOD, 0.21, 0.77)	3.33
5-OH-IMI	42.82	<LOD (<LOD, 1.18, 6.87)	129.09	36.08	<LOD (<LOD, 0.43, 2.63)	19.88	17.13	<LOD (<LOD, <LOD, 1.70)	14.58
6-CNA	1.46	<LOD	1.44	1.65	<LOD	1.92	2.25	<LOD	8.78
Of-IMI	11.19	<LOD (<LOD, <LOD, 5.49)	39.98	3.09	<LOD	105.79	0	<LOD	<LOD
Molar sum of p-NNs and m-NNs (nmol/g)									
∑ACE	96.59	4.37 (1.54, 10.76, 50.47)	603.39	96.08	4.47 (1.74, 10.77, 36.00)	1309.96	99.16	3.93 (2.04, 8.43, 26.14)	106.68
∑DIN	73.97	2.33 (<LOD, 5.93, 20.89)	111.65	61.86	1.28 (<LOD, 4.01, 14.73)	66.5	87.36	2.52 (1.09, 5.24, 15.08)	106.39
∑IMI	48.42	<LOD (<LOD, 9.57, 42.37)	518.94	40.21	<LOD (<LOD, 1.89, 19.21)	383.28	17.70	<LOD (<LOD, <LOD, 6.57)	59.04
∑NNIs	100.00	22.19 (11.24, 40.51, 107.49)	782.02	98.76	22.65 (10.71, 45.32, 119.96)	1386.16	100.00	23.56 (13.07, 38.79, 86.81)	322.95

Note: LOD, limit of detection (ng/mL); ACE, acetamiprid; CLO, clothianidin; CYC, cycloxaprid; DIN, dinotefuran; FLO, flonicamid; FLU, flupyradifurone; IMI, imidacloprid; IMID, Imidaclothiz; NIT, nitenpyram; THIA, thiacloprid; THM, thiamethoxam; N-dm-ACE, N-desmethyl-acetamiprid; DIN-G, 1-methyl-3-(tetrahydro-3-furylmethyl) guanidine; DIN-U, 1-methyl-3-(tetrahydro-3-furylmethyl) urea; 5-OH-IMI, 5-hydroxy-imidacloprid; 6-CNA, 6-chloronicotinic acid; Of-IMI, olefin-imidacloprid; ∑SUL, the molar sum of SUL-α and SUL-β (two isomers of SUL); ∑ACE, the molar sum of ACE and N-dm-ACE; ∑DIN, the molar sum of DIN, DIN-G and DIN-U; ∑IMI, the molar sum of IMI, 5-OH-IMI, and Of-IMI; ∑NNIs, the molar sum of all neonicotinoids.

## Data Availability

The raw data supporting the conclusions of this article will be made available by the authors on request.

## Supplementary Materials

The following supporting information can be downloaded at: https://www.mdpi.com/article/10.3390/toxics13121058/s1, Table S1. Chemical structure, formula, and CAS of detected neonicotinoid insecticides and their metabolites; Table S2. The value of parameters to estimate the daily exposure dose of NNIs; Table S3. The information on eight food groups, Table S4. Estimation of daily exposure dose (EDED; μg/kg/d) of NNIs in children at 7, 10, and 14 years; Table S5. Health risk assessment of NNIs in children at 7, 10, and 14 years by hazard quotient (HQ) and hazard index (HI); Table S6. Associations between dietary food groups and urinary NNIs among school-aged children using linear mixed models; Table S7. Associations between dietary food groups and urinary NNIs among school-aged children who participated in all three follow-ups; Table S8. Associations between dietary food groups and urinary NNIs with high detection frequencies at every single point using multivariable linear models. Table S9. Comparison of urinary NNIs concentrations among children in various studies. Figure S1. Correlations of urinary NNIs concentration among school-aged children.

## Abbreviations

NNIs: neonicotinoid insecticides; nAChRs, nicotinic acetylcholine receptors; IMI, imidacloprid; TDSs, total diet studies; SUL, sulfoxaflor; CYC, cycloxaprid; DIN, dinotefuran; 5-OH-IMI, 5-hydroxy-imidacloprid; N-dm-ACE, N-desmethyl-acetamiprid; SMBCS, Sheyang Mini Birth Cohort Study; BMI, body mass index; SD, standard deviation; ACE, acetamiprid; CLO, clothianidin; FLO, flonicamid; FLU, flupyradifurone; IMID, Imidaclothiz; NIT, nitenpyram; THIA, thiacloprid; THM, thiamethoxam; DIN-G, 1-methyl-3-(tetrahydro-3-furylmethyl) guanidine; DIN-U, 1-methyl-3-(tetrahydro-3-furylmethyl) urea; 6-CNA, 6-chloronicotinic acid; Of-IMI, olefin-imidacloprid; UPLC, ultra-high performance liquid chromatography; HRMS, high-resolution mass spectrometer; QC, quality control; LOD, limit of detection; EDED, estimation of daily exposure dose; HQ, hazard quotient; HI, hazard index; cRfD, chronic reference dose; EPA, Environmental Protection Agency; ADI, acceptable daily dose; MOA, Ministry of Agriculture; CFCT, Chinese Food Composition Tables; LMM, linear mixed model.
