# Supplementary material for: Longitudinal Health Risk Assessment of Neonicotinoid Exposure and Its Association with Dietary Sources in School-Aged Children: A Prospective Cohort Study
Source: Toxics. 2025 Dec 5;13(12):1058. doi: 10.3390/toxics13121058 (PMC12737067; doi:10.3390/toxics13121058)
Supplement: Supplementary file 1 [file toxics-13-01058-s001.zip › toxics-4005023-supplementary.pdf]

Supplemental Material

Longitudinal Health Risk Assessment of Neonicotinoid Exposure and Its Association with Dietary Sources in School-Aged Children: A Prospective Cohort Study

Boya Zhang [1,†], Yiming Dai [1,†], Jiming Zhang [1,*], Zheng Wang [1], Jiayun Ding [1], Xingzu Zhou [1], Xiaojuan Qi [1,2] and Zhijun Zhou [1,*]

[1] MOE Key Laboratory of Public Health Safety, NHC Key Laboratory of Health Technology Assessment, School of Public Health, Fudan University, No. 130 Dong'an Road, Shanghai 200032, China; 22111020059@m.fudan.edu.cn (B.Z.); 20111020031@fudan.edu.cn (Y.D.); 23111020063@m.fudan.edu.cn (Z.W.); 22211020077@m.fudan.edu.cn (J.D.); 24211020091@m.fudan.edu.cn (X.Z.); xjqi@cdc.zj.cn (X.Q.)

[2] Zhejiang Provincial Center for Disease Control and Prevention, No. 3399 Binsheng Road, Hangzhou 310051, China

* Correspondence: zhangjiming@fudan.edu.cn (J.Z.); zjzhou@fudan.edu.cn (Z.Z.)

† These authors contributed equally to this work.

Contents

Table S1 Chemical structure, formula, and CAS of detected neonicotinoid insecticides and their metabolites.

Table S2 The value of parameters to estimate daily exposure dose of NNIs.

Table S3 The information of eight food groups.

Table S4 Estimation of daily exposure dose (EDED; μg/kg/d) of NNIs in children at 7, 10, and 14 years.

Table S5 Health risk assessment of NNIs in children at 7, 10, and 14 years by hazard quotient (HQ) and hazard index (HI).

Table S6 Associations between dietary food groups and urinary NNIs among school-aged children using linear mixed models.

Table S7 Associations between dietary food groups and urinary NNIs among school-aged children who participated in all three follow-ups.

Table S8 Associations between dietary food groups and urinary NNIs with high detection frequencies at each single point using multi-variables linear models.

Table S9 Comparison of urinary NNIs concentrations among children in various studies.

Figure S1 Correlations of urinary NNIs concentration among school-aged children.

Table S1 Chemical structure, formula, and CAS of detected neonicotinoid insecticides and their metabolites.

Analytes	Name	Abbreviation	CAS No.	Formula
**p-NNIs**				
	Acetamiprid	ACE	135410-20-7	$C_{10}H_{11}ClN_4$
	Clothianidin	CLO	210880-92-5	$C_6H_8ClN_5O_2S$
	Cycloxaprid	CYC	1203791-41-6	$C_{14}H_{15}ClN_4O_3$
	Dinotefuran	DIN	165252-70-0	$C_7H_{14}N_4O_3$
	Flonicamid	FLO	158062-67-0	$C_9H_6F_3N_3O$
	Flupyradifurone	FLU	951659-40-8	$C_{12}H_{11}ClF_2N_2O_2$
	Imidacloprid	IMI	105827-78-9	$C_9H_{10}ClN_5O_2$
	Imidaclothiz	IMID	105843-36-5	$C_7H_8ClN_5O_2S$
	Nitenpyram	NIT	150824-47-8	$C_{11}H_{15}ClN_4O_2$
	Thiacloprid	THIA	111988-49-9	$C_{10}H_9ClN_4S$
	Sulfoxaflor	SUL	946578-00-3	$C_{10}H_{10}F_3N_3OS$
	Thiamethoxam	TMX	153719-23-4	$C_8H_{10}ClN_5O_3S$
**m-NNIs**				
Specific metabolites of ACE	N-desmethyl -acetamiprid	N-dm-ACE	190604-92-3	$C_9H_9ClN_4$
Specific metabolites of DIN	1-methyl-3-(tetrahydro-3-furylmethyl) guanidine	DIN-G	457614-32-3	$C_7H_{15}N_3O$
Specific metabolites of DIN	1-methyl-3-(tetrahydro-3-furylmethyl) urea	DIN-U	457614-34-5	$C_7H_{14}N_2O$
Specific metabolites of IMI	5-hydroxy-imidacloprid	5-OH-IMI	380912-09-4	$C_9H_{10}ClN_5O_3$
Nonspecific metabolites	6-chloronicotinic acid	6-CNA	5326-23-8	$C_6H_4ClNO_2$
Specific metabolites of IMI	Olefin-imidacloprid	Of-IMI	115086-54-9	$C_9H_8ClN_5O_2$

Table S2 The value of parameters to estimate daily exposure dose of NNIs.

Analytes	P	cRfD (mg/kg/d)	ADI (mg/kg/d)
ACE	**0.48**	0.057	0.071
CLO	0.596	0.0098	0.097
CYC	0.745	-	-
DIN	0.899	0.02	0.22
FLO	0.51	-	0.07
FLU	**0.33**	-	0.08
IMI	0.133	0.071	0.057
IMID	0.133	-	0.025
NIT	0.46	-	0.53
THIA	0.63	0.004	0.01
∑SUL	**0.51**	-	0.05
THM	0.75	0.006	0.08
N-dm-ACE	0.586	-	-
DIN-G	0.899	-	-
DIN-U	0.899	-	-
5-OH-IMI	**0.31**	-	-
6-CNA	**0.072**	-	-
Of-IMI	**0.27**	-	-

Note: P, neonicotinoid excretion proportion in urine as unchanged species or metabolite); cRfD, chronic reference dose; ADI, acceptable daily intake; ∑SUL, the molar sum of SUL-α and SUL-β (two isomers of SUL); -, no value for reference.

Table S3 The information of eight food groups.

**Foods Group**	**Foods Subgroup**
Cereals	wheat, rice, corn, barley, millet and glutinous millet, others
Vegetables	root vegetables, fresh legume vegetables, solanaceous and gourd vegetables, allium vegetables, tender stems, leaves, and inflorescence vegetables, aquatic vegetables, fresh taro vegetables, wild vegetables
Fruits	pome fruits, stone fruits, berries, citrus fruits, tropical and subtropical fruits, melons
Meat and meat products	pork, beef, lamb, donkey, horse, others
Poultry and poultry products	chicken, duck, goose, turkey, others
Dairy products	liquid milk, milk powder, yogurt, cheese, cream, others
Eggs and egg products	chicken eggs, duck eggs, goose eggs, quail eggs
Aquatic products	fish, shrimp, crab, shellfish, others

Table S4 Estimation of daily exposure dose (EDED; µg/kg/d) of neonicotinoids in children at 7, 10, and 14 years.

Analytes	7-years (n=411)					10-years (n=485)					14-years (n=356)				
	Median	75th	95th	99th	Max	Median	75th	95th	99th	Max	Median	75th	95th	99th	Max
ACE	-	-	0.003	0.025	5.223	-	-	0.008	0.021	0.316	-	-	-	0.024	0.039
CLO	-	0.006	0.047	0.096	0.495	0.013	0.039	0.216	0.620	1.186	0.030	0.078	0.202	0.352	1.381
CYC	-	-	0.028	0.092	0.190	-	0.026	0.181	0.330	2.998	-	-	0.025	0.055	0.105
DIN	-	-	-	-	0.092	-	-	0.009	0.026	0.233	-	-	0.028	0.110	0.310
FLO	-	0.006	0.029	0.067	0.133	-	0.014	0.043	0.162	1.064	-	0.013	0.043	0.129	1.637
FLU	-	-	-	0.008	0.132	-	-	-	0.012	0.026	-	-	-	0.005	0.012
IMI	-	0.015	0.162	0.403	1.665	-	-	0.060	0.168	0.420	-	-	-	0.088	0.158
IMID	-	-	-	0.053	0.304	-	-	-	0.026	0.083	-	-	-	0.034	0.153
NIT	-	0.024	0.103	0.405	2.130	-	0.005	0.035	0.148	0.251	0.015	0.028	0.061	0.152	0.447
THIA	-	-	-	0.007	0.338	-	-	-	0.006	0.016	-	-	-	0.002	0.003
∑SUL	-	-	-	0.087	1.420	-	-	-	0.017	0.375	-	-	-	0.025	0.212
THM	-	-	0.062	0.168	0.506	-	0.031	0.128	0.366	1.026	0.017	0.052	0.162	0.388	1.586
N-dm-ACE	0.027	0.065	0.332	0.757	2.221	0.029	0.071	0.247	1.000	7.247	0.026	0.054	0.195	0.430	0.834
DIN-G	0.002	0.014	0.056	0.160	0.318	-	0.009	0.042	0.095	0.133	0.004	0.010	0.032	0.065	0.084
DIN-U	-	0.003	0.012	0.034	0.237	-	-	0.009	0.033	0.068	0.002	0.004	0.017	0.038	0.093
5-OH-IMI	-	0.068	0.406	1.378	8.105	-	0.023	0.156	0.402	1.060	-	-	0.097	0.320	0.709
6-CNA	-	-	-	0.076	0.329	-	-	-	0.167	0.515	-	-	-	0.077	1.647
Of-IMI	-	-	0.357	0.803	2.855	-	-	-	0.747	6.689	-	-	-	-	-
∑ACE	0.027	0.068	0.338	0.824	5.223	0.030	0.072	0.254	1.002	7.247	0.026	0.055	0.196	0.430	0.834
∑DIN	0.007	0.018	0.067	0.186	0.318	0.004	0.013	0.050	0.100	0.296	0.008	0.018	0.062	0.127	0.473
∑IMI	-	0.156	0.782	2.134	9.770	-	0.033	0.391	1.307	6.689	-	-	0.123	0.369	0.866
∑NNIs	0.177	0.379	1.033	3.384	9.803	0.127	0.292	0.981	2.422	7.667	0.166	0.301	0.701	1.562	3.096

Note: -, no value due to being below the limit of detection. ∑SUL, the molar sum of SUL-α and SUL-β (two isomers of SUL); ∑ACE, the molar sum of ACE and N-dm-ACE; ∑DIN, the molar sum of DIN, DIN-G and DIN-U; ∑IMI, the molar sum of IMI, 5-OH-IMI, and Of-IMI; ∑NNIs, the molar sum of all neonicotinoids.

Table S5 Health risk assessment of neonicotinoids in children at 7, 10, and 14 years by hazard quotient (HQ) and hazard index (HI).

**HQ**	7-years (n=411)					10-years (n=496)					14-years (n=360)				
	Median	75th	95th	99th	Max	Median	75th	95th	99th	Max	Median	75th	95th	99th	Max
ACE	-	-	0.0001	0.0004	0.0916	-	-	0.0002	0.0004	0.0055	-	-	-	0.0004	0.0007
CLO	-	0.0006	0.0048	0.0098	0.0505	0.0013	0.0040	0.0221	0.0633	0.1210	0.0031	0.0079	0.0206	0.0359	0.1410
DIN	-	-	-	-	0.0046	-	-	0.0005	0.0013	0.0117	-	-	0.0014	0.0055	0.0155
FLO	-	0.0001	0.0004	0.0010	0.0019	-	0.0002	0.0006	0.0023	0.0152	-	0.0002	0.0006	0.0018	0.0234
FLU	-	-	-	0.0001	0.0017	-	-	-	0.0002	0.0003	-	-	-	0.0001	0.0001
IMI	-	0.0003	0.0027	0.0067	0.0278	-	-	0.0010	0.0028	0.0070	-	-	-	0.0015	0.0026
IMID	-	-	-	0.0021	0.0122	-	-	-	0.0011	0.0033	-	-	-	0.0014	0.0061
NIT	-	-	0.0002	0.0008	0.0040	-	-	0.0001	0.0003	0.0005	-	0.0001	0.0001	0.0003	0.0008
THIA	-	-	-	0.0018	0.0845	-	-	-	0.0016	0.0041	-	-	-	0.0004	0.0007
∑SUL	-	-	-	0.0018	0.0284	-	-	-	0.0004	0.0075	-	-	-	0.0005	0.0042
THM	-	-	0.0103	0.0280	0.0843	-	0.0052	0.0214	0.0611	0.1710	0.0028	0.0087	0.0270	0.0647	0.2643
**HI**	0.0007	0.0041	0.0188	0.0442	0.2045	0.0036	0.0105	0.0406	0.1117	0.2223	0.0074	0.0186	0.0417	0.0893	0.4054

Note: ∑SUL, the molar sum of SUL-α and SUL-β (two isomers of SUL); -, No value due to being below the limit of detection.

Table S6 Associations between dietary food groups and urinary NNIs among school-aged children using linear mixed models.

	ΣACE			ΣDIN			ΣNNIs		
	β (95%CI)	*p*-value	$p_{sex\text{-}int}$	β(95%CI)	*p*-value	$p_{sex\text{-}int}$	β(95%CI)	*p*-value	$p_{sex\text{-}int}$
**Total**									
Cereals	-0.015 (-0.098, 0.067)	0.714	0.883	-0.155 (-0.255, -0.054)	**0.003**	0.591	-0.042 (-0.100, 0.017)	0.168	0.757
Vegetables	0.059 (-0.142, 0.260)	0.567	**0.107**	0.018 (-0.229, 0.266)	0.884	0.432	0.013 (-0.131, 0.158)	0.856	**0.089**
Fruits	0.043 (-0.001, 0.088)	0.056	0.974	0.011 (-0.044, 0.065)	0.706	**0.123**	0.041 (0.009, 0.073)	**0.012**	0.768
Meat and meat products	-0.036 (-0.225, 0.153)	0.709	0.996	0.163 (-0.069, 0.395)	0.168	0.464	-0.011 (-0.146, 0.125)	0.879	0.409
Poultry and poultry products	-0.118 (-0.273, 0.037)	0.137	0.212	-0.250 (-0.441, -0.060)	**0.010**	0.424	-0.048 (-0.159, 0.064)	0.402	0.946
Dairy products	0.005 (-0.074, 0.084)	0.900	0.861	-0.010 (-0.107, 0.087)	0.833	0.310	0.023 (-0.034, 0.080)	0.423	0.587
Eggs and egg products	-0.247 (-0.568, 0.074)	0.132	0.381	-0.205 (-0.599, 0.190)	0.310	0.403	-0.233 (-0.464, -0.002)	**0.048**	0.205
Aquatic products	0.050 (-0.068, 0.169)	0.406	0.700	0.070 (-0.076, 0.215)	0.349	0.277	0.019 (-0.066, 0.105)	0.654	0.445
**Boys**									
Cereals	-0.011 (-0.094, 0.073)	0.801		-0.150 (-0.253, -0.047)	**0.005**		-0.045 (-0.106, 0.016)	0.151	
Vegetables	0.055 (-0.149, 0.258)	0.599		0.014 (-0.240, 0.267)	0.916		0.008 (-0.142, 0.158)	0.914	
Fruits	0.044 (-0.001, 0.089)	0.059		0.010 (-0.046, 0.066)	0.736		0.042 (0.009, 0.075)	**0.013**	
Meat and meat products	-0.013 (-0.205, 0.178)	0.893		0.163 (-0.075, 0.402)	0.179		0.007 (-0.135, 0.148)	0.924	
Poultry and poultry products	-0.120 (-0.277, 0.037)	0.135		-0.249 (-0.444, -0.054)	**0.013**		-0.047 (-0.163, 0.069)	0.425	
Dairy products	0.013 (-0.067, 0.093)	0.746		-0.012 (-0.112, 0.087)	0.806		0.029 (-0.030, 0.088)	0.329	
Eggs and egg products	-0.254 (-0.578, 0.070)	0.125		-0.188 (-0.592, 0.216)	0.362		-0.247 (-0.486, -0.008)	**0.043**	
Aquatic products	0.046 (-0.073, 0.166)	0.449		0.071 (-0.078, 0.220)	0.351		0.014 (-0.075, 0.102)	0.763	
**Girls**									
Cereals	-0.007 (-0.104, 0.089)	0.882		-0.114 (-0.231, 0.003)	0.056		-0.022 (-0.090, 0.045)	0.515	
Vegetables	0.259 (0.070, 0.448)	**0.007**		-0.113 (-0.344, 0.118)	0.338		0.172 (0.040, 0.304)	**0.011**	
Fruits	0.038 (-0.017, 0.093)	0.174		0.075 (0.009, 0.142)	**0.026**		0.048 (0.010, 0.086)	**0.014**	
Meet and meat products	-0.070 (-0.330, 0.190)	0.598		-0.004 (-0.321, 0.312)	0.978		0.062 (-0.120, 0.243)	0.504	
Poultry and poultry products	0.025 (-0.142, 0.192)	0.771		-0.141 (-0.344, 0.062)	0.175		-0.053 (-0.170, 0.063)	0.370	
Dairy products	0.012 (-0.102, 0.127)	0.834		-0.106 (-0.245, 0.033)	0.136		-0.015 (-0.095, 0.065)	0.705	
Eggs and egg products	-0.072 (-0.411, 0.268)	0.680		0.045 (-0.368, 0.458)	0.832		-0.051 (-0.288, 0.186)	0.673	
Aquatic products	0.011 (-0.131, 0.152)	0.884		-0.062 (-0.235, 0.110)	0.478		0.074 (-0.025, 0.173)	0.141	

Note: ∑ACE, the molar sum of ACE and N-dm-ACE; ∑DIN, the molar sum of DIN, DIN-G and DIN-U; ∑NNIs, the molar sum of all neonicotinoids. Models were adjusted by sex, maternal education, maternal occupational type, residence, annual household income, passive smoking, childhood BMI z-score.

Table S7 Associations between dietary food groups and urinary NNIs among school-aged children who participated in all three follow-ups.

	ΣACE			ΣDIN			ΣNNIs		
	β (95%CI)	*p*-value	*p* $_{sex\text{-}int}$	β(95%CI)	*p*-value	*p* $_{sex\text{-}int}$	β(95%CI)	*p*-value	*p* $_{sex\text{-}int}$
**Total**									
Cereals	0.003 (-0.105, 0.112)	0.951	0.392	-0.134 (-0.269, 0.001)	0.052	**0.170**	-0.033 (-0.107, 0.042)	0.390	**0.181**
Vegetables	-0.045 (-0.322, 0.232)	0.750	**0.018**	0.020 (-0.330, 0.369)	0.913	0.980	0.063 (-0.127, 0.252)	0.518	**0.053**
Fruits	0.015 (-0.046, 0.076)	0.633	0.825	0.015 (-0.061, 0.091)	0.701	**0.072**	0.039 (-0.003, 0.080)	0.070	0.910
Meat and meat products	-0.022 (-0.292, 0.249)	0.874	0.282	0.310 (-0.026, 0.646)	0.071	0.196	0.058 (-0.127, 0.242)	0.541	0.375
Poultry and poultry products	-0.115 (-0.351, 0.121)	0.340	0.568	-0.511 (-0.803, -0.219)	**0.001**	**0.052**	-0.056 (-0.218, 0.105)	0.495	0.730
Dairy products	-0.033 (-0.125, 0.060)	0.490	0.587	-0.015 (-0.130, 0.100)	0.800	0.914	-0.001 (-0.064, 0.062)	0.977	0.489
Eggs and egg products	-0.208 (-0.688, 0.271)	0.395	0.337	-0.500 (-1.102, 0.101)	0.104	0.243	-0.369 (-0.698, -0.040)	**0.028**	**0.169**
Aquatic products	0.018 (-0.130, 0.166)	0.812	0.377	0.030 (-0.156, 0.215)	0.752	0.703	-0.001 (-0.103, 0.100)	0.977	0.505
**Boys**									
Cereals	0.007 (-0.097, 0.111)	0.894		-0.133 (-0.271, 0.006)	0.061		-0.036 (-0.111, 0.038)	0.337	
Vegetables	-0.068 (-0.335, 0.199)	0.618		0.010 (-0.348, 0.368)	0.956		0.048 (-0.143, 0.240)	0.620	
Fruits	0.020 (-0.038, 0.079)	0.500		0.012 (-0.066, 0.090)	0.765		0.045 (0.003, 0.087)	**0.036**	
Meat and meat products	0.040 (-0.220, 0.299)	0.766		0.301 (-0.045, 0.648)	0.089		0.092 (-0.094, 0.278)	0.334	
Poultry and poultry products	-0.112 (-0.337, 0.113)	0.328		-0.520 (-0.817, -0.224)	**0.001**		-0.054 (-0.216, 0.107)	0.510	
Dairy products	-0.015 (-0.104, 0.073)	0.736		-0.018 (-0.136, 0.100)	0.766		0.006 (-0.057, 0.069)	0.855	
Eggs and egg products	-0.221 (-0.682, 0.240)	0.348		-0.507 (-1.124, 0.110)	0.108		-0.360 (-0.689, -0.031)	**0.033**	
Aquatic products	0.006 (-0.137, 0.148)	0.938		0.039 (-0.152, 0.230)	0.691		-0.018 (-0.120, 0.084)	0.734	
**Girls**									
Cereals	0.087 (-0.079, 0.253)	0.306		0.018 (-0.178, 0.214)	0.856		0.064 (-0.045, 0.174)	0.251	
Vegetables	0.406 (0.135, 0.677)	**0.004**		0.068 (-0.256, 0.392)	0.682		0.329 (0.151, 0.507)	**<0.001**	
Fruits	-0.010 (-0.106, 0.086)	0.835		0.129 (0.017, 0.241)	**0.025**		0.038 (-0.026, 0.101)	0.246	
Meet and meat products	0.260 (-0.151, 0.671)	0.216		-0.063 (-0.547, 0.421)	0.799		0.198 (-0.074, 0.470)	0.154	
Poultry and poultry products	-0.030 (-0.291, 0.231)	0.822		-0.096 (-0.402, 0.211)	0.541		0.001 (-0.172, 0.174)	0.992	
Dairy products	0.035 (-0.139, 0.210)	0.692		-0.027 (-0.234, 0.179)	0.795		-0.048 (-0.164, 0.068)	0.416	
Eggs and egg products	0.108 (-0.495, 0.712)	0.726		0.117 (-0.592, 0.826)	0.746		-0.045 (-0.444, 0.355)	0.827	
Aquatic products	-0.122 (-0.371, 0.126)	0.336		0.093 (-0.199, 0.385)	0.533		-0.062 (-0.227, 0.102)	0.460	

Note: ∑ACE, the molar sum of ACE and N-dm-ACE; ∑DIN, the molar sum of DIN, DIN-G and DIN-U; ∑NNIs, the molar sum of all neonicotinoids. Models were adjusted by sex, maternal education, maternal occupational type, residence, annual household income, passive smoking, childhood BMI z-score.

Table S8 Associations between dietary food groups and urinary NNIs with high detection frequencies at each single point using multi-variables linear models.

	ΣACE		ΣDIN		ΣNNIs		CLO		NIT	
	β (95%CI)	*p*-value	β(95%CI)	*p*-value	β(95%CI)	*p*-value	β(95%CI)	*p*-value	β(95%CI)	*p*-value
**7 years**										
Cereals	-0.11 (-0.37, 0.15)	0.411	-0.16 (-0.46, 0.14)	0.309	-0.06 (-0.24, 0.11)	0.476	-	-	-	-
Vegetables	0.21 (-0.26, 0.68)	0.380	-0.22 (-0.75, 0.32)	0.428	0.16 (-0.15, 0.47)	0.317	-	-	-	-
Fruits	0.10 (-0.10, 0.29)	0.333	0.00 (-0.22, 0.22)	0.979	0.17 (0.05, 0.30)	**0.007**	-	-	-	-
Meat and meat products	-0.19 (-0.75, 0.38)	0.520	0.31 (-0.33, 0.95)	0.346	0.19 (-0.18, 0.57)	0.316	-	-	-	-
Poultry and poultry products	-0.39 (-0.90, 0.12)	0.133	-0.12 (-0.70, 0.47)	0.691	0.23 (-0.11, 0.57)	0.178	-	-	-	-
Dairy products	0.10 (-0.14, 0.34)	0.415	0.04 (-0.23, 0.32)	0.754	0.13 (-0.02, 0.29)	0.098	-	-	-	-
Eggs and egg products	-0.37 (-1.26, 0.52)	0.417	-0.32 (-1.33, 0.69)	0.535	-0.49 (-1.08, 0.10)	0.103	-	-	-	-
Aquatic products	0.12 (-0.13, 0.37)	0.351	0.15 (-0.13, 0.44)	0.298	0.16 (-0.01, 0.33)	0.061	-	-	-	-
**10 years**										
Cereals	0.04 (-0.12, 0.19)	0.647	-0.06 (-0.25, 0.12)	0.502	0.01 (-0.11, 0.12)	0.921	0.05 (-0.13, 0.22)	0.585	-	-
Vegetables	-0.15 (-0.44, 0.14)	0.319	0.06 (-0.30, 0.43)	0.732	-0.07 (-0.30, 0.16)	0.556	-0.10 (-0.45, 0.24)	0.565	-	-
Fruits	0.07 (-0.01, 0.14)	0.099	-0.01 (-0.11, 0.08)	0.816	0.04 (-0.02, 0.10)	0.199	0.07 (-0.02, 0.16)	0.146	-	-
Meat and meat products	-0.05 (-0.43, 0.33)	0.798	-0.29 (-0.76, 0.18)	0.226	-0.25 (-0.55, 0.06)	0.111	0.02 (-0.43, 0.47)	0.919	-	-
Poultry and poultry products	-0.12 (-0.33, 0.09)	0.259	-0.23 (-0.49, 0.02)	0.072	-0.07 (-0.24, 0.09)	0.392	0.06 (-0.18, 0.30)	0.621	-	-
Dairy products	-0.02 (-0.15, 0.12)	0.783	-0.15 (-0.32, 0.01)	0.068	-0.00 (-0.11, 0.10)	0.975	-0.09 (-0.25, 0.06)	0.250	-	-
Eggs and egg products	-0.10 (-0.58, 0.39)	0.700	-0.06 (-0.66, 0.54)	0.847	-0.22 (-0.60, 0.17)	0.268	-0.32 (-0.89, 0.24)	0.265	-	-
Aquatic products	0.04 (-0.11, 0.20)	0.577	0.09 (-0.09, 0.28)	0.322	-0.02 (-0.14, 0.10)	0.785	0.01 (-0.17, 0.19)	0.885	-	-
**14 years**										
Cereals	0.00 (-0.11, 0.11)	0.977	-0.08 (-0.21, 0.06)	0.282	-0.04 (-0.11, 0.03)	0.280	0.03 (-0.13, 0.19)	0.737	0.09 (-0.04, 0.21)	0.184
Vegetables	0.28 (-0.06, 0.62)	0.113	0.29 (-0.14, 0.72)	0.191	0.13 (-0.09, 0.36)	0.245	0.10 (-0.40, 0.59)	0.702	-0.14 (-0.53, 0.25)	0.486
Fruits	0.03 (-0.02, 0.08)	0.201	0.02 (-0.04, 0.09)	0.485	0.03 (-0.00, 0.06)	0.093	0.01 (-0.07, 0.08)	0.886	0.04 (-0.01, 0.10)	0.136
Meet and meat products	0.00 (-0.23, 0.23)	0.984	0.18 (-0.11, 0.47)	0.220	0.05 (-0.10, 0.20)	0.551	0.08 (-0.26, 0.41)	0.654	-0.04 (-0.30, 0.22)	0.780
Poultry and poultry products	0.04 (-0.23, 0.31)	0.771	-0.09 (-0.43, 0.26)	0.623	-0.06 (-0.24, 0.12)	0.528	-0.03 (-0.43, 0.36)	0.864	-0.03 (-0.34, 0.28)	0.851
Dairy products	-0.01 (-0.10, 0.09)	0.891	0.04 (-0.08, 0.16)	0.543	0.00 (-0.06, 0.07)	0.909	0.05 (-0.09, 0.19)	0.472	-0.04 (-0.15, 0.08)	0.530
Eggs and egg products	-0.46 (-0.93, 0.00)	0.051	-0.51 (-1.10, 0.07)	0.088	-0.21 (-0.52, 0.10)	0.176	0.09 (-0.59, 0.76)	0.806	-0.19 (-0.73, 0.34)	0.478
Aquatic products	0.02 (-0.43, 0.47)	0.942	0.64 (0.08, 1.20)	**0.027**	0.04 (-0.26, 0.34)	0.779	-0.42 (-1.08, 0.23)	0.206	0.43 (-0.09, 0.94)	0.105

Note: ∑ACE, the molar sum of ACE and N-dm-ACE; ∑DIN, the molar sum of DIN, DIN-G and DIN-U; ∑NNIs, the molar sum of all neonicotinoids. Models were adjusted by sex, maternal education, maternal occupational type, residence, annual household income, passive smoking, childhood BMI z-score.

Table S9 Comparison of urinary NNIs concentrations among children in various studies.

Reference	Sampling time	Age (year)	Country/Region	Sample size	ACE		CLO		CYC		DIN		FLO		FLU		IMI		IMID		NIT	
					Conc.	DF, %	Conc.	DF, %	Conc.	DF, %	Conc.	DF, %	Conc.	DF, %	Conc.	DF, %	Conc.	DF, %	Conc.	DF, %	Conc.	DF, %
Our study [a]	2016	7	Jiangsu, China	411	< LOD (0.01)	7.54	< LOD (0.06)	30.66	< LOD (0.10)	8.76	< LOD (0.40)	0.97	< LOD (0.08)	29.44	< LOD (0.04)	3.65	< LOD (0.06)	28.95	< LOD (0.04)	2.43	< LOD (0.06)	45.26
	2019	10		485	<LOD	14.02	0.43	65.15	<LOD	30.93	<LOD	8.66	<LOD	44.33	<LOD	3.51	<LOD	16.7	<LOD	2.27	<LOD	31.96
	2023	14		356	<LOD	4.78	0.98	77.81	<LOD	6.74	<LOD	10.39	<LOD	33.43	<LOD	1.40	<LOD	4.78	<LOD	1.40	0.39	73.88
(Oya et al., 2021) [a]	2015.6-2016.8	16-23m	Aichi, Japan	1036	<LOD	37.3	<LOD	32.7	/	/	<LOD	45.8	/	/	/	/	<LOD	40.4	/	/	/	/
(Su et al., 2025) [b]	2015-2016	6-11	U.S.	326	<LOD	0.6	<LOD	7.1	/	/	/	/	/	/	/	/	<LOD	5.8	/	/	/	/
		12-19		309	<LOD	0.3	<LOD	8.2	/	/	/	/	/	/	/	/	<LOD	6.0	/	/	/	/
(Zhao et al., 2022) [b]	2017.9	8-11	Shenzhen, China	305	0.01	95.1	0.19	93.4	/	/	1.64	97.7	/	/	/	/	0.13	95.7	/	/	/	/
(Lu et al., 2023) [a]	2017-2020	7	Shandong, China	380	0.007	82.9	0.488	98.9	/	/	0.193	97.6	/	/	/	/	0.454	99.7	/	/	<LOD	36.6
(Wu et al., 2024) [a]	2019.11	11.3-16.1	Chongqing, China	524	2.28	100.0	5.08	99.8	/	/	2.67	92.1	1.48	80.1	/	/	3.57	99.8	4.66	82.0	2.81	92.8
(Yang et al., 2024) [a]	2019-2020	7-11	Shanghai, China	442	<LOD	1.4	0.72	53.6	/	/	<LOD	3.8	/	/	/	/	<LOD	4.1	<LOD	0	<LOD	7.2

Continued from Table S9

Reference	Age (year)	Country/Region	THIA		ΣSUL		THM		N-dm-ACE		DIN-G		DIN-U		5-OH-IMI		6-CNA		Of-IMI	
			Conc.	DF, %	Conc.	DF, %	Conc.	DF, %	Conc.	DF, %	Conc.	DF, %	Conc.	DF, %	Conc.	DF, %	Conc.	DF, %	Conc.	DF, %
Our study [a]	7	Jiangsu, China	< LOD (0.05)	2.92	< LOD	2.19	< LOD (0.05)	24.09	0.88	96.35	0.10	54.99	< LOD (0.03)	46.47	<LOD (0.10)	42.82	< LOD (0.10)	1.46	< LOD (0.43)	11.19
	10		<LOD	4.12	<LOD	1.24	<LOD	48.66	0.91	96.08	<LOD	49.28	<LOD	20.41	<LOD	36.08	<LOD	1.65	<LOD	3.09
	14		<LOD	1.40	<LOD	1.12	0.79	52.53	0.82	99.16	0.19	71.63	0.12	70.51	<LOD	17.13	<LOD	2.25	<LOD	0
(Oya et al., 2021) [a]	16-23m	Aichi, Japan	<LOD	21.8	/	/	<LOD	41.1	<LOD	14.1	/	/	/	/	/	/	/	/	/	/
(Su et al., 2025) [b]	6-11	U.S.	<LOD	0.6	/	/	/	/	<LOD	46.9	/	/	/	/	<LOD	17.5	/	/	/	/
	12-19		<LOD	0.6	/	/	/	/	<LOD	37.6	/	/	/	/	<LOD	20.1	/	/	/	/
(Zhao et al., 2022) [b]	8-11	Shenzhen, China	0.002	94.8	/	/	0.21	99.7	/	/	/	/	/	/	/	/	/	/	/	/
(Lu et al., 2023) [a]	7	Shandong, China	0.010	77.6	/	/	0.074	95.5	0.977	100.0	/	/	/	/	/	/	0.967	97.9	/	/
(Wu et al., 2024) [a]	11.3-16.1	Chongqing, China	2.64	98.7	1.43	98.7	3.51	100.0	34.27	98.0	/	/	/	/	/	/	/	/	/	/
(Yang et al., 2024) [a]	7-11	Shanghai, China	<LOD	0	/	/	/	/	1.64	57.0	/	/	/	/	<LOD	0	/	/	/	/

Note: Conc, concentration; DF, detection frequency; LOD, limit of detection; "/" means not included in the analytes. [a]. The concentration of NNIs were creatinine-adjusted (μg/g Cr). [b]. The concentration of NNIs were not adjusted (μg/L).

Reference

Lu, Z. P., Hu, Y., Tse, L. A., Yu, J. X., Xia, Z. N., Lei, X. N., Zhang, Y., Shi, R., Tian, Y., & Gao, Y. (2023). Urinary neonicotinoid insecticides and adiposity measures among 7-year--old children in northern China: A cross-sectional study. *International Journal of Hygiene and Environmental Health*, *251*. https://doi.org/ARTN 11418810.1016/j.ijheh.2023.114188

Oya, N., Ito, Y., Ebara, T., Kato, S., Ueyama, J., Aoi, A., Nomasa, K., Sato, H., Matsuki, T., Sugiura-Ogasawara, M., Saitoh, S., & Kamijima, M. (2021). Cumulative exposure assessment of neonicotinoids and an investigation into their intake-related factors in young children in Japan. *Science of the Total Environment*, *750*. https://doi.org/ARTN 14163010.1016/j.scitotenv.2020.141630

Su, Q., Luo, J. Z., Zhou, Y., Liu, M., Zeng, S. H., Li, Y., & Gao, J. Y. (2025). Sex steroid hormones mediate the association between neonicotinoids and obesity among children and adolescents. *Ecotoxicology and Environmental Safety*, *289*. https://doi.org/ARTN 11770810.1016/j.ecoenv.2025.117708

Wu, X., Liu, Q., Li, Y., Yue, M., Su, Q., Luo, J. Z., Li, Y., Zeng, S. H., & Gao, J. Y. (2024). Urinary neonicotinoid concentrations and obesity: A cross-sectional study among Chinese adolescents. *Environmental Pollution*, *345*. https://doi.org/ARTN 12351610.1016/j.envpol.2024.123516

Yang, Z. C., Wang, Y. P., Tang, C. X., Han, M. H., Wang, Y., Zhao, K., Liu, J. Q., Tian, J. C., Wang, H. X., Chen, Y., & Jiang, Q. W. (2024). Urinary neonicotinoids and metabolites are associated with obesity risk in Chinese school children. *Environment International*, *183*. https://doi.org/ARTN 10836610.1016/j.envint.2023.108366

Zhao, Y., Zhu, Z., Xiao, Q. R., Li, Z. H., Jia, X. H., Hu, W. T., Liu, K. C., & Lu, S. Y. (2022). Urinary neonicotinoid insecticides in children from South China: Concentrations, profiles and influencing factors. *Chemosphere*, *291*. https://doi.org/ARTN 13293710.1016/j.chemosphere.2021.132937

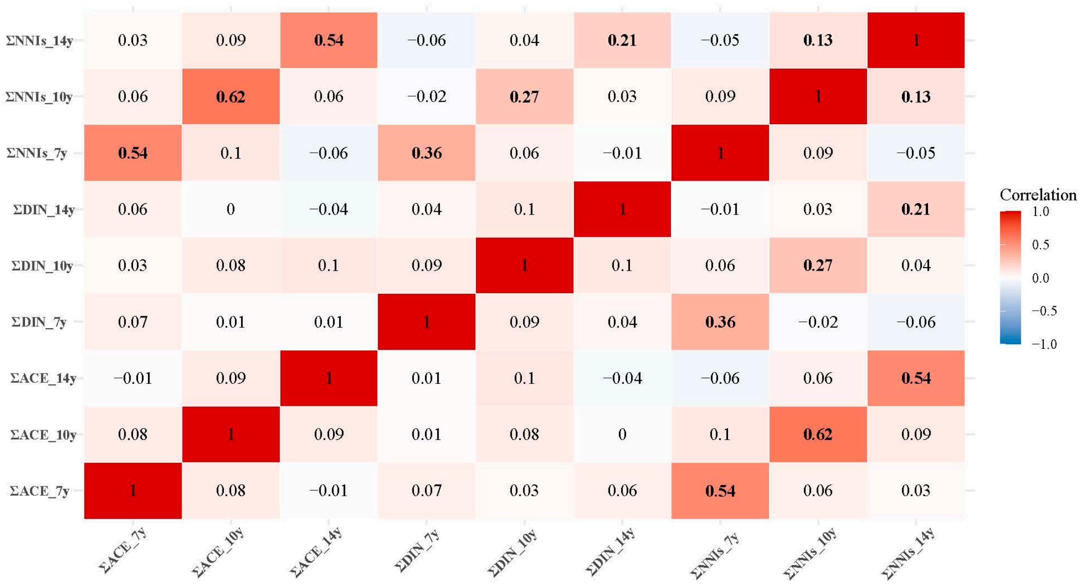

Figure S1 Correlations of urinary NNIs concentration among school-aged children. Bold fonts represent significant correlations (p-value <0.05).
